# Supplementary material for: Rigidity enhances a magic-number effect in polymer phase separation
Source: Nat Commun. 2020 Mar 25;11:1561. doi: 10.1038/s41467-020-15395-6 (PMC7096466; doi:10.1038/s41467-020-15395-6)
Supplement: Supplementary file 1 — Supplementary Information [file 41467_2020_15395_MOESM1_ESM.pdf]

Rigidity enhances a magic-number effect in polymer phase separation
Supplementary Note 1

Bin Xu,[1] Guanhua He,[2] Benjamin G. Weiner,[1] Pierre Ronceray,[3] Yigal Meir,[4] Martin C. Jonikas,[2] and Ned S. Wingreen[2,5]

[1] *Department of Physics, Princeton University*
[2] *Department of Molecular Biology, Princeton University*
[3] *Princeton Center for Theoretical Science, Princeton University*
[4] *Department of Physics, Ben Gurion University of the Negev*
[5] *Lewis-Sigler Institute for Integrative Genomics, Princeton University*

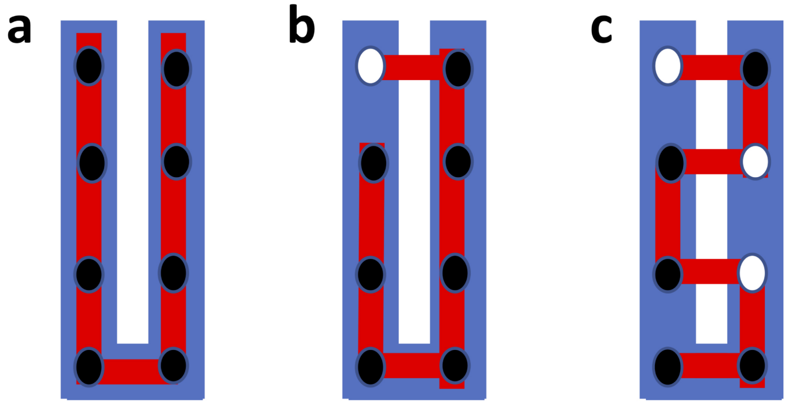

Supplementary Figure 1. Schematic illustration of the U-shaped system A_8:B_{8U}. Black dots correspond to specific bonds and white dots are monomers not in bonds. Only one (randomly picked) bond can form when the flexible polymer crosses the gap (white space in the middle).

Cluster size in a null-model with no interactions

In the fully-bonded regime, the average cluster sizes in magic-number systems are lower than those in the non-magic-number systems (Fig. 1**g-l,m,o**). However, average cluster sizes in magic-number systems also become very high at high concentrations. This increase of cluster size occurs because at high polymer concentrations the limited free volume for dimers favors condensation. Is the resulting average cluster size larger than one would expect in a noninteracting system due to accidental overlaps? To estimate the effect of accidental overlaps, we consider a null model with no interactions other than the single-occupancy condition for each species. As shown in Fig. 1**m,o**, the average cluster sizes in the magic-number systems are close to those in this null model, supporting our conclusion that magic-number systems do not experience enhanced clustering due to the specific interaction.

There is no magic-number effect for weak interactions

In the heat maps of the magic-number systems (Fig. 1**h,k**) there is a "bump" in the phase boundary, *i.e.*, the cluster size initially increases, and then decreases with specific bond energy. This occurs because in the weak-interaction regime thermal fluctuations lead to free binding sites. The presence of these free binding sites precludes the magic-number effect; indeed, the system resembles non-magic-number cases and, similarly, the phase boundary moves to lower concentration with increasing interaction strength. However, as the interaction strength is increased further, all possible bonds form and the magic-number effect takes over, shifting the phase boundary back to higher concentration. The net result is the observed non-monotonic behavior ("bump") of the phase boundary with increasing specific bond energy for the magic-number systems.

The magnitude of the magic-number effect increases with valence

Valence is a key parameter in both natural and engineered two-component systems that form specific bonds[1–4]. It is therefore natural to ask how valence influences the magic-number effect. To theoretically address this question, we studied systems composed of two species of flexible polymers A_n:B_m over a range of valences (Supplementary Fig. 2). We observed that non-magic-number systems (A_{n-1}:B_n) display more clustering than the corresponding magic-number systems A_n:B_n, despite the higher valence of the latter. Specifically, average cluster size at low concentrations decreases by a factor of 2 from A_3:B_4 to A_4:B_4, 5 from A_5:B_6 to A_6:B_6, and 10 from A_7:B_8 to A_8:B_8 (Supplementary Fig. 2 insets). In Supplementary Fig. 2, we also compared the phase boundaries, *i.e.*, the concentrations at which large clusters begin to form (details in following paragraphs). Note that for each pair, the valence is lower for the non-magic-number system, which competes with the magic-number effect. Indeed, for the lowest-valence case, A_3:B_4 has a similar phase boundary to A_4:B_4 (Supplementary Fig. 2**a**). By contrast, for the higher-valence systems, the phase boundaries in the regime of strong interactions occur at substantially higher concentrations for the magic-number systems (Supplementary Fig. 2**b,c**). This confirms that higher valence increases the magnitude of the magic-number effect. Note that at lower interaction energies, the presence of unbonded monomers means magic- and non-magic-number systems behave similarly, which accounts for the "bump" in the phase boundary for A_6:B_6 and A_8:B_8.

We determined approximate phase diagrams for the A_3:B_4, A_4:B_4, A_5:B_6, A_6:B_6, A_7:B_8, and A_8:B_8 systems based on fluctuations of cluster size. For each system and concentration, we first increased the non-specific bond from 0 to 0.1 k_BT in 0.005 k_BT increments, keeping the specific bond energy at 0 k_BT. Then the specific bond energy was increased from 0 to 11 k_BT in 0.04 k_BT increments, while the non-specific bond energy was kept at 0.1 k_BT. Each step of annealing was simulated with at least 50,000 Monte-Carlo steps (*i.e.* proposed moves) per monomer. While increasing the specific bond energy, we recorded the average cluster sizes when the specific bond energy was an integral multiple of 0.4 k_BT. This simulation was independently repeated 100 times to compute cluster-size fluctuations, *i.e.* the standard deviation over 100 simulations of the average cluster size in each simulation.

For each specific bond energy, we expect the peak of cluster-size fluctuations to closely indicate the phase boundary. Therefore, we fit the standard deviation of average cluster size versus concentration with a Gaussian and took the peak to be the concentration corresponding to the phase transition. Cluster-size fluctuations corresponding to different specific energies were fitted independently. The smoothed curves in Supplementary Fig. 2 were obtained via cubic spline, with additional fictitious data points included in the fit (but not shown) to induce a vertical slope for the highest specific bond energies, consistent with the saturation of all bonds.

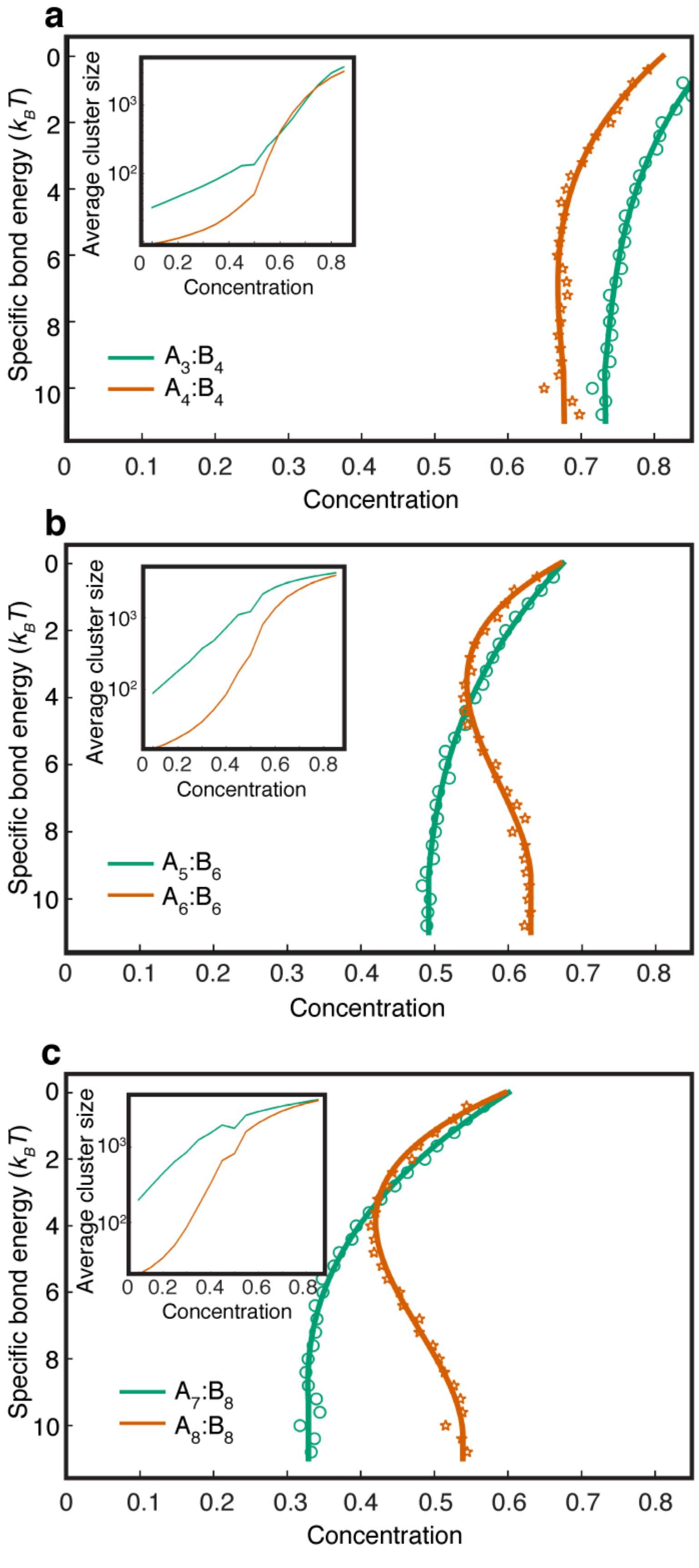

Supplementary Figure 2. Phase boundaries of **a** A_3:B_4 and A_4:B_4, **b** A_5:B_6 and A_6:B_6, **c** A_7:B_8 and A_8:B_8 systems, obtained from fluctuation analysis of mean cluster sizes for different specific bond energies. Insets: average cluster size of the systems in the main panels versus monomer concentrations, for specific bond energy = 10 $k_\mathrm{B}T$. Parameters: non-specific bond energy = 0.1 $k_\mathrm{B}T$, A:B monomer ratio = 1.

Three-dimensional model

Simulations were performed on a cubic lattice of $20 \times 20 \times 20$ grid points (or "sites") with periodic boundary conditions. In the model, polymers with different shapes occupy several connected (nearest neighbor) sites such that each monomer occupies one site. There are two species of polymer in each simulation, denoted as A_n or B_n, with n being the number of monomers in one polymer. A monomer of A and a monomer of B form a specific bond when they occupy the same site in the 3D lattice; no more than one A-monomer and no more than one B-monomer can occupy a site.

The A polymers are considered to be "flexible," such that any configuration of connected nearest-neighbor sites is allowed. The B polymers ($\mathrm{B}_{8\mathrm{R}}$) are rigid $2 \times 2 \times 2$ cubes.

Systems with only these specific interactions have a very weak effective surface tension between condensed and dilute phases, which prevents formation of dense droplets. Motivated by the existence of weak non-specific interactions between polymers (*e.g.*, due to hydrophobicity), we add a small non-specific interaction between all nearest neighbor monomers as described below, which increases the surface tension between phases and results in denser droplets.

We performed Markov-Chain Monte-Carlo simulations using the Metropolis algorithm. Briefly, in each simulation step we randomly propose a move of the configuration. The move is always accepted if it reduces system energy, and accepted with probability $\mathrm{e}^{-(E_\mathrm{f}-E_\mathrm{i})/k_\mathrm{B}T}$, where E_f and E_i are the final and initial energies, if the move increases system energy. Three categories of moves are proposed: single-flexible-polymer moves, single-rigid moves, and two-species joint moves. Single-flexible-polymer moves are standard lattice-polymer local moves: the end-point move, the corner move, and the reptation move. Single-rigid moves consist of one-step translations in the six cardinal directions. In the regime of strong specific bonds, the two species are typically held together by multiple specific bonds, which leads to dynamical freezing. To enable the system to better explore configuration space, we include a two-species joint move such that connected clusters of polymers are translated together, without breaking any specific bonds. To obtain thermalized ensembles, we follow a simulated annealing procedure: we keep $k_\mathrm{B}T$ constant and gradually increase bond strength. We increase the specific bond energy in 0.1 $k_\mathrm{B}T$ increments, from 0 to the final bond strength (2, 3, 4, or 5 $k_\mathrm{B}T$), while the non-specific bond energy is kept at 0.1 $k_\mathrm{B}T$. The system thermalizes over 15,000 Monte-Carlo steps (*i.e.* proposed moves) per monomer, then results are averaged over the subsequent 15,000 steps.

Supplementary Fig. 3 demonstrates that the magic-number effect is present in three dimensions. Snapshots (Supplementary Fig. 3**a-c**) show the magic-number system A_8:$\mathrm{B}_{8\mathrm{R}}$ dominated by dimers, whereas the non-magic-number cases A_7:$\mathrm{B}_{8\mathrm{R}}$ and A_9:$\mathrm{B}_{8\mathrm{R}}$ form large clusters at the same concentration and binding energy. Heatmaps of mean cluster size confirm that the formation of large clusters is suppressed in the magic-number system (Supplementary Fig. 3**d-f**), and Supplementary Fig. 3**h** quantifies the prevalence of dimers.

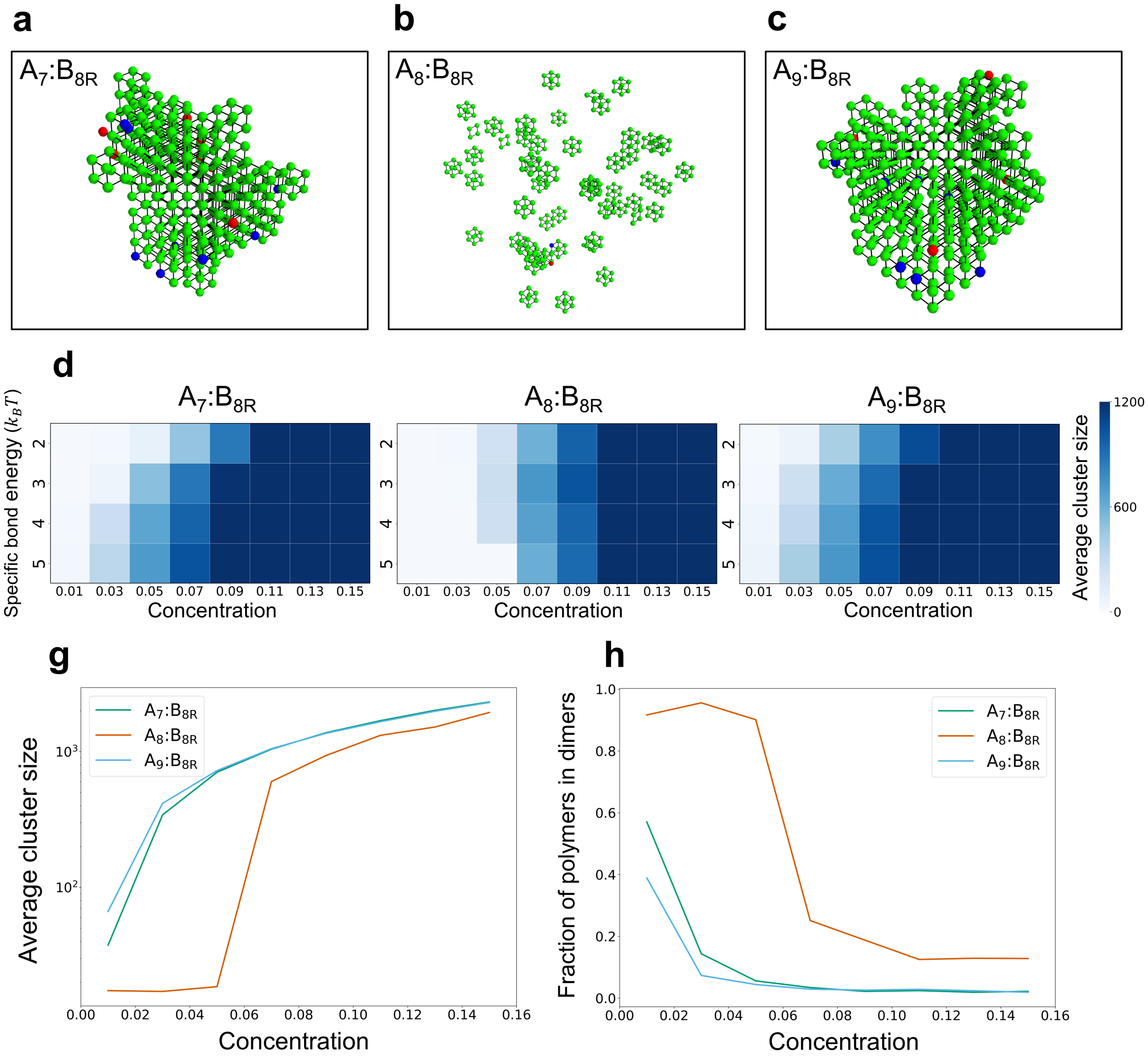

Supplementary Figure 3. The magic-number effect is present in three dimensions. **a-c** Snapshots of simulations on a 3D cubic lattice of **a** A_7:B_{8R}, **b** A_8:B_{8R}, and **c** A_9:B_{8R} systems, where A_n denotes flexible polymers of valence n, and B_{8R} denotes rigid $2 \times 2 \times 2$ cubes. The flexible polymers (A_n) are red, the rigid cubes (B_{8R}) are blue, and specific bonds where these overlap are green. Parameters: specific bond energy $= 5\ k_{\mathrm{B}}T$, non-specific bond energy $= 0.1\ k_{\mathrm{B}}T$, A:B monomer ratio $= 1$, monomer concentration of each species $= 0.05$. **d-f** Heat maps of average cluster size as functions of total monomer concentration (of each species) and strength of specific bonds for systems in **a-c**. The ratio of A:B monomer concentration is equal to one, and the non-specific bond energy $= 0.1\ k_{\mathrm{B}}T$. **g** Average cluster size for A_7:B_{8R}, A_8:B_{8R}, and A_9:B_{8R} systems with specific bond energy $5\ k_{\mathrm{B}}T$, i.e. horizontal cuts through **d-f**. **h** Fraction of polymers in dimers for A_7:B_{8R}, A_8:B_{8R}, and A_9:B_{8R} systems with specific bond energy $5\ k_{\mathrm{B}}T$.

Phase separation versus percolation

The biological condensates under consideration are phase-separated droplets and behave as liquids. However, some systems where polymers bond and form connected clusters undergo a homogeneous sol-gel transition. Gels have different physical properties than liquids, which would presumably have implications for biological function. How can we distinguish between phase separation and homogeneous gelation in our two-component multivalent systems?

Within our mean-field theory, the system clearly undergoes phase separation as a first-order phase transition: the free energy is non-convex and the order parameter (density) has a discontinuity, both hallmarks of a first-order transition. Specifically, the non-convex profile of the free energy implies that the ground state of the system in the thermodynamic limit necessarily consists of two phases for all concentrations lying between the points where the tie line is tangent to the free-energy profile, and the densities of the two phases are those corresponding the points of tangency (cf. Supplementary Fig. 8). (Note that although we use mean cluster size as an order parameter throughout this work, here we use density because it exhibits more spatial variation in the finite lattice simulations and facilitates theoretical calculations.) In the Monte Carlo simulations, local density analysis suggests that the macroscopic clusters are phase-separated droplets rather than percolation clusters arising from homogeneous gelation. First, visual inspection of the interacting system (Supplementary Fig. 4**a**) reveals that as the system concentration increases, the cluster or clusters become larger but not denser. In the noninteracting system, in which "bonds" between polymers of different types arise purely from accidental overlaps, this is not the case (Supplementary Fig. 4**b**). Instead, these systems form large clusters as a simple consequence of percolation. Supplementary Fig. 4**c** quantifies this difference in terms of the average local density inside the cluster at concentrations where macroscopic clusters are present. To define a local density, we calculate the number of neighbors for every monomer inside a cluster that includes more than half the proteins. (Note that because every site can be occupied by two monomers, the density ranges from 0 to 2.) This quantity is averaged over monomers, and then over Monte Carlo samples. In the interacting system, the local density is quite stable as the system concentration grows. By contrast, the noninteracting ("gel-like") percolation clusters become progressively denser as the concentration grows. This confirms the visual impression from snapshots that the interacting system undergoes phase separation rather than homogeneous gelation. As the macroscopic clusters we observe are always internally connected by a network of specific bonds, we conclude that our system represents a case of gelation driven by phase separation[5,6].

Finally, it should be noted that suddenly increasing the bond strength (a"quench") can trap the system in a kinetically-arrested state far from equilibrium. This metastable state resembles a network of fibers (Supplementary Fig. 5**a**), which is easily distinguished from the equilibrium droplets (Supplementary Fig. 5**b**) by visual inspection.

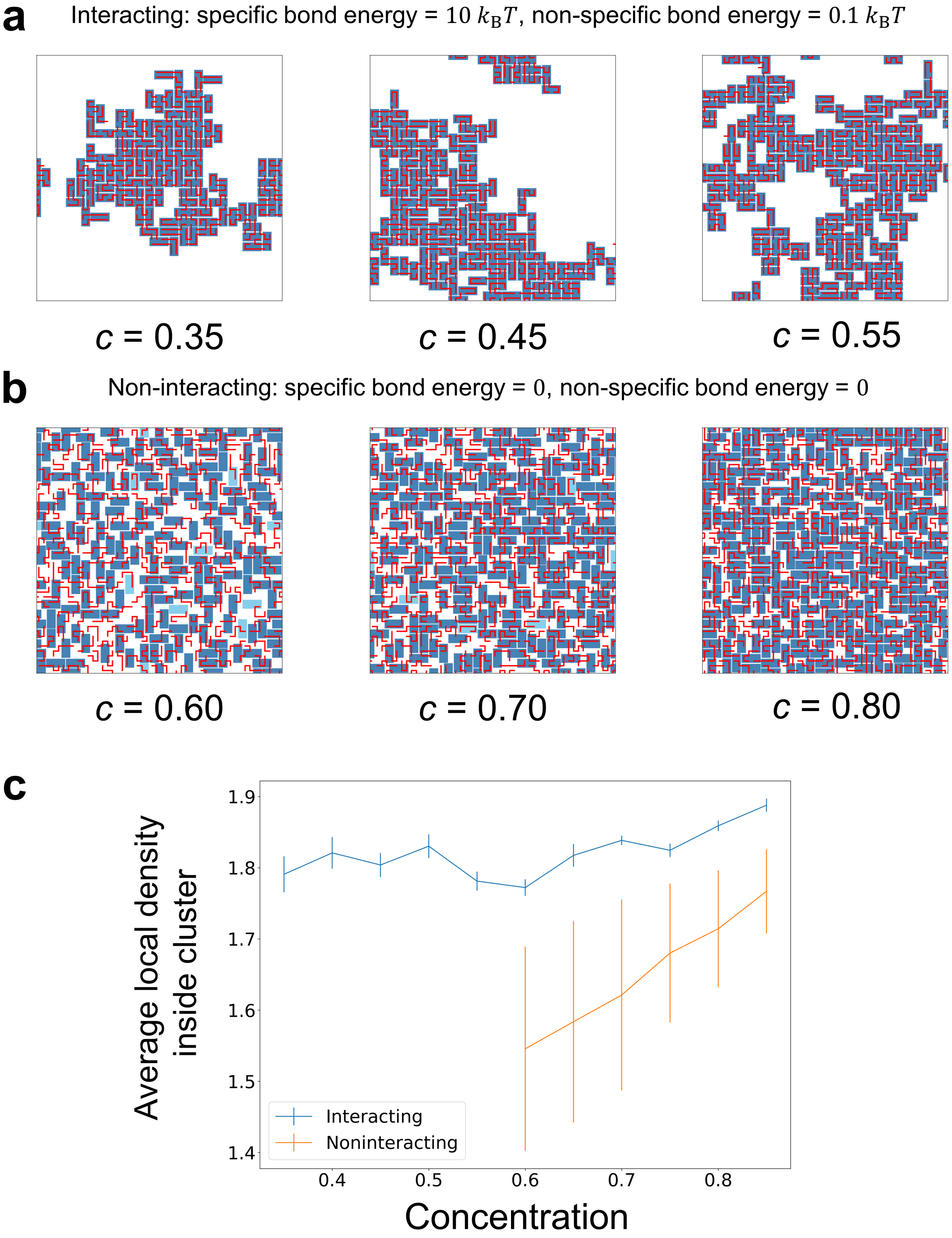

Supplementary Figure 4. Clusters are phase-separated droplets rather than artifacts of percolation. **a** Snapshots of simulations of A_7:$\mathrm{B}_{8\mathrm{R}}$ where cluster formation is driven by specific bonds. Parameters: specific bond energy = 10 $k_{\mathrm{B}}T$, non-specific bond energy = 0.1 $k_{\mathrm{B}}T$, A:B monomer ratio = 1. **b** Snapshots of simulations of A_7:$\mathrm{B}_{8\mathrm{R}}$ where cluster formation is due to random percolation. Parameters: specific bond energy = 0 $k_{\mathrm{B}}T$, non-specific bond energy = 0 $k_{\mathrm{B}}T$, A:B monomer ratio = 1. **c** The local density inside macroscopic clusters, averaged over all monomers in the cluster then averaged over independent Monte Carlo samples (mean $\pm$ SD of Monte Carlo samples).

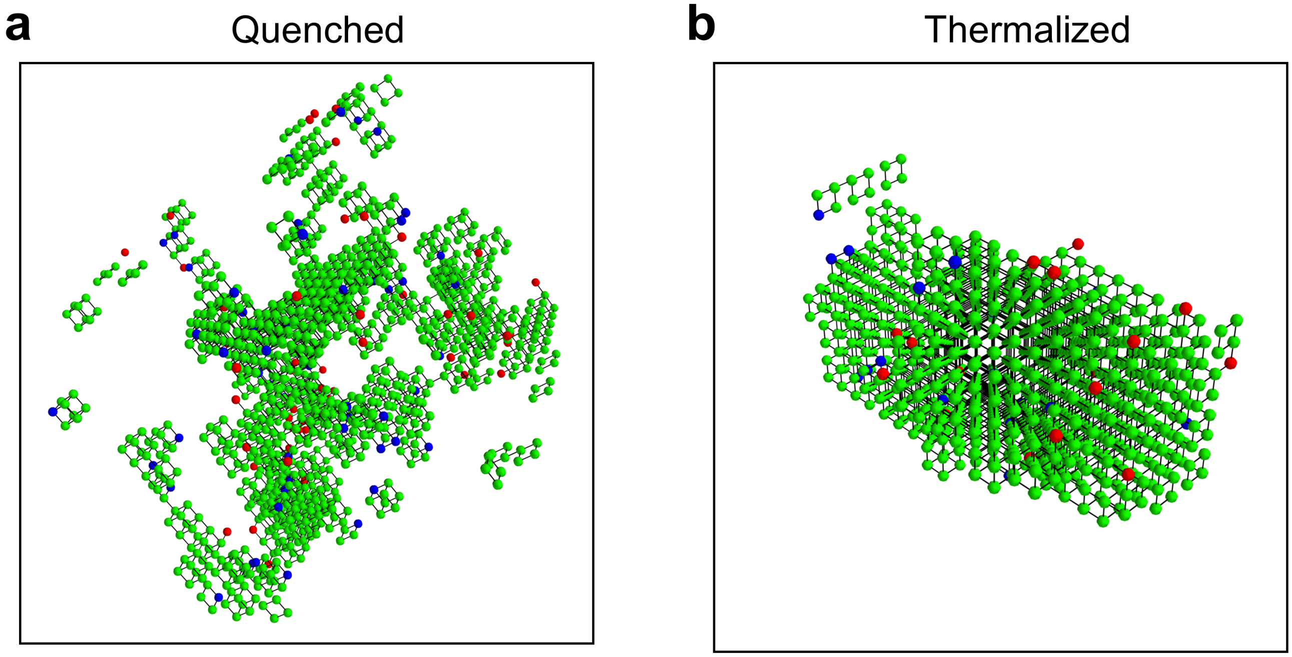

Supplementary Figure 5. Clusters are phase-separated droplets at equilibrium rather than kinetically-arrested states. **a** Snapshot of simulation of A_7:B_{8R} after a temperature quench where specific bond energy increased from $0 \to 5\ k_BT$. The flexible polymers (A_n) are red, the rigid cubes (B_{8R}) are blue, and specific bonds where these overlap are green. Parameters: monomer concentration of each species=0.15, non-specific bond energy $= 0.1\ k_BT$, A:B monomer ratio $= 1$. **b** Snapshot of simulation with same parameters as **a**, but after simulated annealing to a specific bond energy of $5\ k_BT$.

The role of non-specific interactions

The magic-number effect is due to a competition between translational and conformational entropy in the regime of strong specific bond formation. However, our simulations also include non-specific interactions, which lead to more realistic droplets with a higher density and surface tension (see **Methods**). How do such non-specific interactions influence the magic-number effect? First, Supplementary Fig. 6 shows that non-specific interactions alone do not lead to a magic-number effect. With the energy of specific bonds set to zero, no large clusters form, and the magic number case A_8:B_{8R} (Supplementary Fig. 6**b,e**) shows no suppression in cluster size compared to the non-magic number cases A_7:B_{8R} and A_9:B_{8R}. Supplementary Fig. 7 shows the dependence of cluster formation on non-specific interactions for a range of specific bond energies. Weakening the non-specific interactions has two effects: 1) the system must reach higher concentrations before large clusters form and 2) the clusters are less dense. However, this does not change the tendency of magic-number systems to form dimers rather than large clusters. Indeed, the mean cluster size as a function of concentration reveals a magic-number effect even in the absence of non-specific interactions (Supplementary Fig. 7**c**). Taken together, Supplementary Fig. 6 and 7 demonstrate that non-specific interactions are neither sufficient nor necessary for the existence of the magic-number effect.

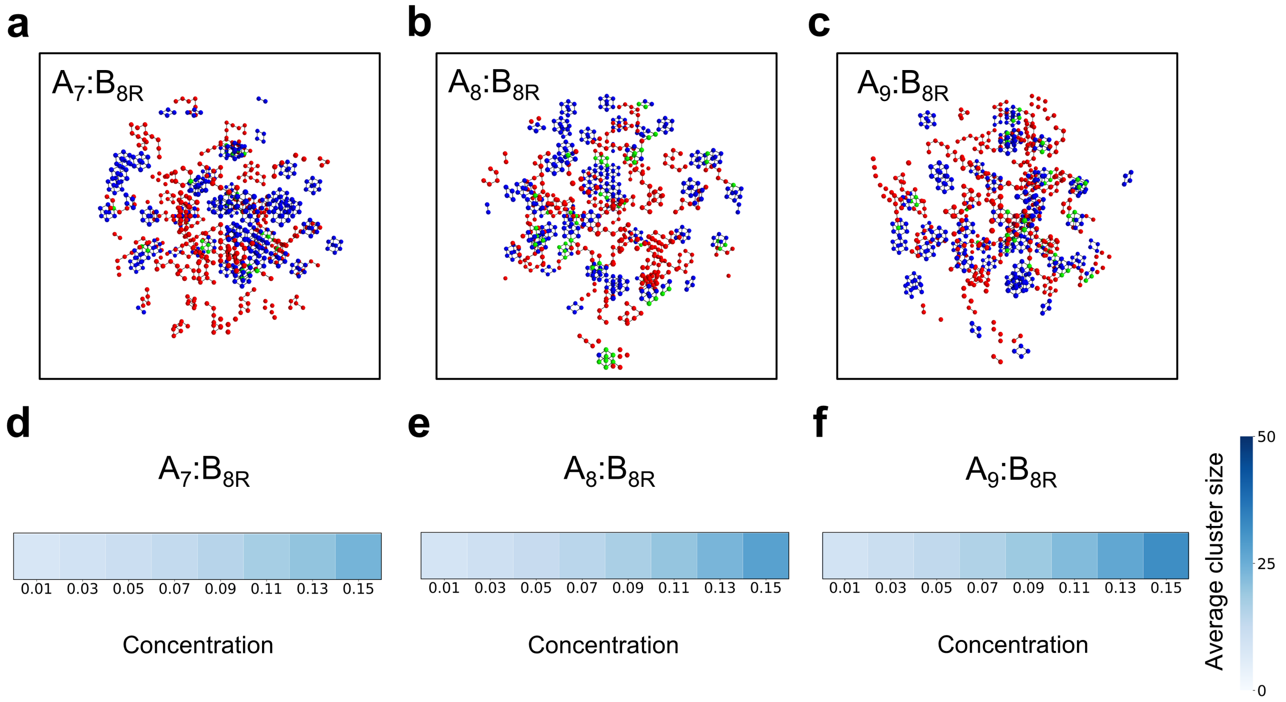

Supplementary Figure 6. Specific bonds are necessary for the magic-number effect. **a-c** Snapshots of simulations on a 3D cubic lattice of **a** A_7:B_{8R}, **b** A_8:B_{8R}, and **c** A_9:B_{8R} systems. The flexible polymers (A_n) are red, the rigid cubes (B_{8R}) are blue, and specific bonds where these overlap are green. Parameters: specific bond energy $= 0$ k_BT, non-specific bond energy $= 0.1$ k_BT, A:B monomer ratio $= 1$, monomer concentration of each species $= 0.05$. **d-f** Heat maps of average cluster size as functions of total monomer concentration (of each species) for systems in **a-c**. The ratio of A:B monomer concentration is equal to one, the non-specific bond energy $= 0.1$ k_BT, and the specific bond energy $= 0$ k_BT.

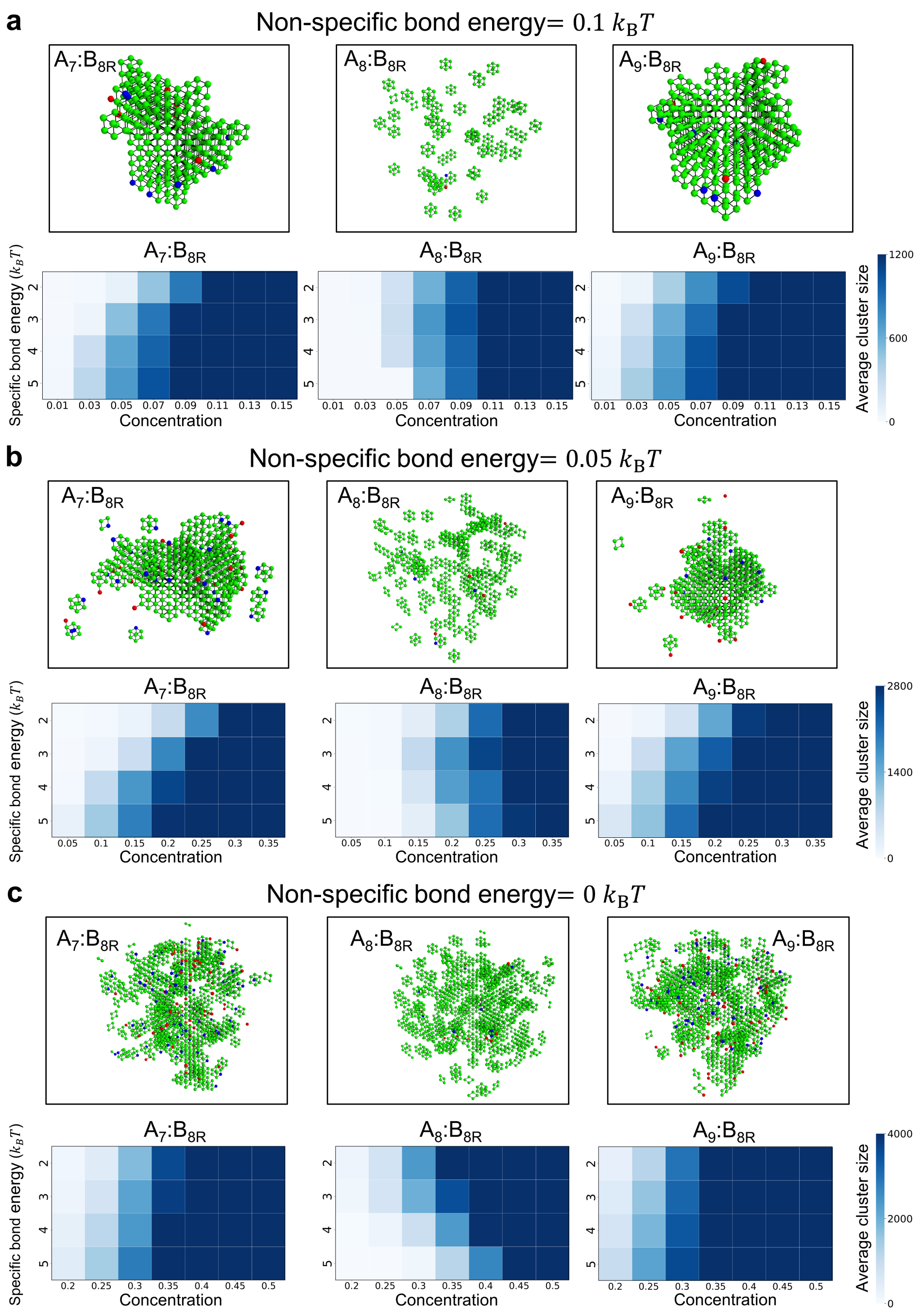

Supplementary Figure 7. The magic-number effect does not require non-specific interactions. **a, Top** Snapshots of simulations on a 3D cubic lattice of (*Left*) A_7:B_{8R}, (*Middle*) A_8:B_{8R}, and (*Right*) A_9:B_{8R} systems. The flexible polymers (A_n) are red, the rigid cubes (B_{8R}) are blue, and specific bonds where these overlap are green. Parameters: specific bond energy $= 5\ k_{\mathrm{B}}T$, non-specific bond energy $= 0.1\ k_{\mathrm{B}}T$, A:B monomer ratio = 1, monomer concentration of each species = 0.05. **a, Bottom** Heat maps of average cluster size as functions of total monomer concentration (of each species) for systems in **a, Top** at different specific bond energies. The ratio of A:B monomer concentration is equal to one and the non-specific bond energy $= 0.1\ k_{\mathrm{B}}T$. **b, Top** Same as **a, Top**, but for non-specific bond energy $= 0.05\ k_{\mathrm{B}}T$ and monomer concentration of each species = 0.10. **b, Bottom** Same as **a, Bottom**, but for non-specific bond energy $= 0.05\ k_{\mathrm{B}}T$. **c, Top** Same as **a, Top**, but for non-specific bond energy $= 0\ k_{\mathrm{B}}T$ and monomer concentration of each species = 0.20. **c, Bottom** Same as **a, Bottom**, but for non-specific bond energy $= 0\ k_{\mathrm{B}}T$.

Flory-Huggins theory: a brief review

Flory-Huggins theory[7] provides a simple analytical treatment of phase separation in a polymer-solvent system. The theory estimates both the configurational entropy of the polymers and the enthalpy of polymer-solvent interaction within a mean-field approximation. For strong enough polymer-solvent repulsion, the resulting free-energy density is a non-convex function of the concentration, which implies phase separation.

To briefly review, the Flory-Huggins model considers a lattice on which solvents and polymers occupy sites. Each solvent molecule or monomer occupies one site and all sites are taken to be singly occupied. We consider a system with N_p polymers, each with L monomers, and N_s solvent molecules. On the lattice with M sites in total, each site has z neighbors.

In order to compute the configurational entropy, we need to estimate the number of configurations in the fully mixed state. Assuming all solvent molecules are identical, they make no contribution to the number of configurations. (Assuming solvent molecules to be distinguishable only contributes a factorial constant, which does not affect the final phase diagram.)

Amongst the M lattice sites, we first select N_p points on the lattice to be the "head" of each polymer, thus the number of head configurations is:

$$w_\mathrm{head} = \frac{M!}{(M-N_\mathrm{p})!N_\mathrm{p}!}. \tag{S1}$$

For each of the other monomers of the polymer ("body"), it can choose among $(z-1)$ sites if the sites are unoccupied. Given the single-occupancy constraint, the number of positions that a "body" monomer can be chosen to occupy is estimated as $(z-1)\frac{M-N_\mathrm{occ}}{M}$, where N_occ is the total number of occupied lattice sites before this monomer is placed. Then the number of configurations for all "body" monomers is:

$$w_\mathrm{body} = \left(\frac{z-1}{M}\right)^{N_\mathrm{p}(L-1)} \frac{(M-N_\mathrm{p})!}{(M-N_\mathrm{p}L)!}. \tag{S2}$$

Thus the total number of configurations is:

$$W = w_\mathrm{head}w_\mathrm{body} = \left(\frac{z-1}{M}\right)^{N_\mathrm{p}(L-1)} \frac{M!}{N_\mathrm{p}!(M-N_\mathrm{p}L)!}. \tag{S3}$$

For simplicity, it is conventional in Flory-Huggins theory to express the free energy of the well-mixed state relative to a state in which the polymers and solvents are fully separated. In this reference state, the number of configurations is given by Eq. (S3) with $M = N_\mathrm{p}L$:

$$W_0 = \left(\frac{z-1}{N_\mathrm{p}L}\right)^{N_\mathrm{p}(L-1)} \frac{(N_\mathrm{p}L)!}{N_\mathrm{p}!}. \tag{S4}$$

The fractional increase of the number of configurations with respect to the reference state is

$$\frac{W}{W_0} = \left(\frac{N_\mathrm{p}L}{M}\right)^{N_\mathrm{p}(L-1)} \frac{M!}{(M-N_\mathrm{p}L)!(N_\mathrm{p}L)!}. \tag{S5}$$

Using Stirling's approximation, we find the entropy change due to mixing:

$$S_\mathrm{mix} = k_\mathrm{B}\log\frac{W}{W_0} \approx -k_\mathrm{B}\left[(M-N_\mathrm{p}L)\log(1-c) + N_\mathrm{p}\log c\right], \tag{S6}$$

where $c = N_\mathrm{p}L/M$ is the monomer concentration.

Within mean-field theory, the change of enthalpy density, or equivalently internal energy density, upon mixing is:

$$\frac{\Delta U}{Mk_\mathrm{B}T} = \chi c(1-c), \tag{S7}$$

where χ is an interaction parameter, and we express energies in units of the thermal energy $k_\mathrm{B}T$. The change in free-energy density is thus

$$\Delta f \equiv \frac{\Delta F}{Mk_\mathrm{B}T} = \frac{\Delta U - T\Delta S}{Mk_\mathrm{B}T} = \frac{c}{L}\log c + (1-c)\log(1-c) + \chi(1-c)c. \tag{S8}$$

This Flory-Huggins free-energy density is a non-convex function of concentration c if χ is large. The total free energy in the non-convex region is minimized by the system separating into two coexisting phases with concentrations at the two common tangent points of a line touching the free-energy curve from below.

Dimer versus condensate theory for magic-number systems

Here, we generalize the Flory-Huggins theory to systems with two polymer species in addition to the solvent. We first focus on the magic-number condition in which the polymers have the same valency L, and restrict our attention to the case with equal monomer concentrations, with each species having N_p polymers. In the regime of strong specific bonds between the two polymers, all monomers of the two species are considered to be in bonds, and the internal energy is simply a fixed constant, which we neglect. We therefore consider each site in the lattice to be occupied either by two monomers, one from each polymer species, or by solvent.

i. System with two flexible polymer species (A_L:B_L)

We model the two-species system separately in two regimes: the high concentration ("dense") regime, dominated by a condensate, and the low concentration ("dilute") regime, dominated by dimers.

In the dense regime, the number of configurations of the fully-bonded system is the product of the number of configurations of the two independent species, times the probability that the subset of sites occupied by the monomers of one species exactly matches the subset of sites occupied by monomers of the other species:

$$W_\mathrm{dense} = W_1 W_2 P_\mathrm{match}, \tag{S9}$$

where W_1 and W_2 are the total number of lattice configurations for each of the two species considered separately. To compute W_1 and W_2, we slightly modify Eq. (S3): the translational degrees of freedom are still assumed to contribute

$$\left(\frac{1}{M}\right)^{N_\mathrm{p}(L-1)} \frac{M!}{N_\mathrm{p}!(M-N_\mathrm{p}L)!}, \tag{S10}$$

while the number of different self-avoiding polymer conformations with a given head position, *aka* the polymer conformation factor C_L, is exactly computed numerically. The total number of configurations is therefore

$$W_1 = W_2 = \left(\frac{1}{M}\right)^{N_\mathrm{p}(L-1)} \frac{M!}{N_\mathrm{p}!(M-N_\mathrm{p}L)!} C_L^{N_\mathrm{p}}. \tag{S11}$$

Numerical values of some C_Ls are:

L	2	3	4	5	6	7	8
C_L	4	12	36	100	284	780	2172

We adopt a mean-field theory for the matching probability, *i.e.*, we consider one species to occupy its complete subset of sites, and then take the probability for the i-th monomer of the other species to fall within the same subset of sites to be

$$P^{(i)}_\mathrm{match} = \frac{\text{Sites in the matching region \& not already occupied}}{\text{Sites not already occupied}} = \frac{N_\mathrm{p}L-(i-1)}{M-(i-1)}, \tag{S12}$$

so that,

$$P_\mathrm{match} = \prod_i P^{(i)}_\mathrm{match} = \frac{(N_\mathrm{p}L)!(M-N_\mathrm{p}L)!}{M!}. \tag{S13}$$

The number of configurations in the dense case is therefore:

$$W_\mathrm{dense} = \frac{M!(N_\mathrm{p}L)!}{N_\mathrm{p}!^2(M-N_\mathrm{p}L)!M^{2N_\mathrm{p}(L-1)}} C_L^{2N_\mathrm{p}}. \tag{S14}$$

We find the free-energy density relative to the state of zero entropy to be:

$$\begin{aligned} f_\mathrm{dense} &= \frac{-TS_\mathrm{dense}}{Mk_\mathrm{B}T} = \frac{-k_\mathrm{B}T\ln W_\mathrm{dense}}{Mk_\mathrm{B}T} \\ &= \frac{2c}{L}\log\left(\frac{c}{L}\right) - c\log(c) + (1-c)\log(1-c) + \frac{2c}{L}(L-1) - \frac{2c}{L}\log C_L. \end{aligned} \tag{S15}$$

In the dilute regime, we model polymers as fully bonded dimers. The translational degrees of freedom can be approximated by the same expression as in Eq. (S10) now applied to dimers as effectively a single species. Regarding

polymer conformations within a dimer, we can exactly numerically compute the number of different ways of forming one dimer with a given head position of one of its polymer types, *aka* the dimer degeneracy D_L. (Note that $D_L = \sum_j d_{L,j}^2$, where $d_{L,j}$ is the number of conformations of a single polymer of length L occupying a specific set j of lattice sites.) The total number of configurations is

$$W_{\text{dilute}} = \left(\frac{1}{M}\right)^{N_{\text{p}}(L-1)} \frac{M!}{N_{\text{p}}!(M - N_{\text{p}}L)!} D_L^{N_{\text{p}}}, \tag{S16}$$

and the free-energy density relative to a state of zero entropy is

$$f_{\text{dilute}} = \frac{-TS_{\text{dilute}}}{Mk_{\text{B}}T} = \frac{-k_{\text{B}}T \ln W_{\text{dilute}}}{Mk_{\text{B}}T} = \frac{c}{L} \log\left(\frac{c}{L}\right) + (1-c)\log(1-c) + \frac{c}{L}(L-1) - \frac{c}{L}\log(D_L). \tag{S17}$$

Numerical values of some D_Ls are:

L	2	3	4	5	6	7	8
D_L	8	24	120	264	1144	2392	9960

To obtain the free-energy density at an arbitrary concentration c, we simply take the minimum of the dense and dilute estimates of the free energy, *i.e.*,

$$f(c) = \min\left[f_{\text{dense}}(c), f_{\text{dilute}}(c)\right]. \tag{S18}$$

ii. Systems with one flexible polymer species and one rigid species A_8:B_{8R} *and* A_8:B_{8U}

When one species is a rigid shape, the dimer-versus-condensate theory still applies, but with several modifications. One modification is that the polymer that forms the rigid shape has limited flexibility. Therefore, for W_2 in Eq. (S11) in the dense regime, we replace $C_8 = 2172$ by $C_{8R} = 4$, yielding

$$f_{\text{dense}} = \frac{2c}{L}\log\left(\frac{c}{L}\right) - c\log(c) + (1-c)\log(1-c) + \frac{2c}{L}(L-1) - \frac{c}{L}[\log(C_8) + \log(C_{8R})]. \tag{S19}$$

In the dilute regime for the A_8:$B_{8R/U}$ system, the number of ways to form dimers with a given center of mass is reduced from $D_8 = 9960$ to $D_{8R} = 112$ and $D_{8U} = 8$ in Eq. (S16), so that

$$f_{\text{dilute}} = \frac{c}{L}\log\left(\frac{c}{L}\right) + (1-c)\log(1-c) + \frac{c}{L}(L-1) - \frac{c}{L}\log(D_{8R/U}). \tag{S20}$$

Oligomer-versus-condensate theory for non-magic-number systems (A_{L_1}:B_{L_2})

For systems with two flexible species of polymers with different valencies L_1 and L_2, we can similarly extend the dimer-versus-condensate theory. In the dilute regime, as the system can no longer form fully saturated dimers, it forms fully bonded oligomers composed of multiple polymers of each species. For simplicity, we consider the case when L_1 and L_2 are co-prime, which includes our simulations with $L_1 = L_2 - 1$. The smallest oligomer in this case has L_1L_2 monomers of each species. We focus on systems with equal monomer concentrations in the strongly interacting limit, so that all monomers form specific bonds.

The dense regime is similar to that in the magic-number system, but with the two species having different valencies L_1 and L_2. The number of configurations is

$$W_{\text{dense}} = W_{1,L_1} W_{2,L_2} P_{\text{match}} \tag{S21}$$

where, similar to Eq. (S11),

$$W_{1,L_1} = \left(\frac{1}{M}\right)^{N_{\text{p1}}(L_1-1)} \frac{M!}{N_{\text{p1}}!(M - N_{\text{p1}}L_1)!} C_{L_1}^{N_{\text{p1}}}, \tag{S22}$$

$$W_{2,L_2} = \left(\frac{1}{M}\right)^{N_{\text{p2}}(L_2-1)} \frac{M!}{N_{\text{p2}}!(M - N_{\text{p2}}L_2)!} C_{L_2}^{N_{\text{p2}}}. \tag{S23}$$

Following Eq. (S13), we have

$$P_{\text{match}} = \frac{(cM)!(M - cM)!}{M!}, \tag{S24}$$

where $c = \frac{N_{\mathrm{p}1}L_1}{M} = \frac{N_{\mathrm{p}2}L_2}{M}$ is the concentration of each monomer type. Then the free-energy density is:

$$f_{\mathrm{dense}} = \frac{c}{L_1}\log\left(\frac{c}{L_1}\right) + \frac{c}{L_2}\log\left(\frac{c}{L_2}\right) - c\log(c) + (1-c)\log(1-c) + c\left(\frac{L_1-1}{L_1} + \frac{L_2-1}{L_2} - \frac{\log C_{L_1}}{L_1} - \frac{\log C_{L_2}}{L_2}\right). \quad \text{(S25)}$$

In the dilute regime, the system is dominated by oligomers that occupy L_1L_2 lattice sites. Since an oligomer is typically much larger than a dimer in the magic-number systems, it is impractical to exactly numerically compute the oligomer degeneracy, *i.e.*, the number of ways to form a fully-bonded oligomer with $N_1 = L_2$ polymers of the first species and $N_2 = L_1$ polymers of the second species. As a rough approximation, we assume that the dominating configurations form the simplest compact shape, *i.e.*, an $L_1 \times L_2$ rectangle. We then use Eq. (S11) to estimate the number of configurations of each species within an $L_1 \times L_2$ rectangle (so that $M \to L_1L_2$):

$$\begin{aligned} D_1 &= \left(\frac{1}{L_1L_2}\right)^{L_2(L_1-1)} \frac{(L_1L_2)!}{L_2!} C_{L_1}^{L_2}, \\ D_2 &= \left(\frac{1}{L_1L_2}\right)^{L_1(L_2-1)} \frac{(L_1L_2)!}{L_1!} C_{L_2}^{L_1}. \end{aligned} \quad \text{(S26)}$$

The resulting expression for the total number of configurations in the dilute case is then similar to Eq. (S16). Specifically, there are $\left(\frac{1}{M}\right)^{N_{\mathrm{olig}}(L_1L_2-1)} \frac{M!}{N_{\mathrm{olig}}!(M-N_{\mathrm{olig}}L_1L_2)!}$ center-of-mass configurations, where $N_{\mathrm{olig}} = cM/(L_1L_2)$ is the number of oligomers. Each oligomer has a degeneracy $D_{\mathrm{olig}} = 2D_1D_2$, where the factor of 2 comes from the two possible orientations of the oligomer rectangle, and D_1 and D_2 are the number of possible configurations of the two species within the rectangle. Then the total number of configurations is:

$$W_{\mathrm{dilute}} = \left(\frac{1}{M}\right)^{N_{\mathrm{olig}}(L_1L_2-1)} \frac{M!}{N_{\mathrm{olig}}!(M-N_{\mathrm{olig}}L_1L_2)!} D_{\mathrm{olig}}^{N_{\mathrm{olig}}}, \quad \text{(S27)}$$

and the free energy is

$$f_{\mathrm{dilute}} = \frac{c}{L_1L_2}\log\left(\frac{c}{L_1L_2}\right) + (1-c)\log(1-c) + \frac{c}{L_1L_2}(L_1L_2-1) - \frac{c}{L_1L_2}\log(2D_1D_2). \quad \text{(S28)}$$

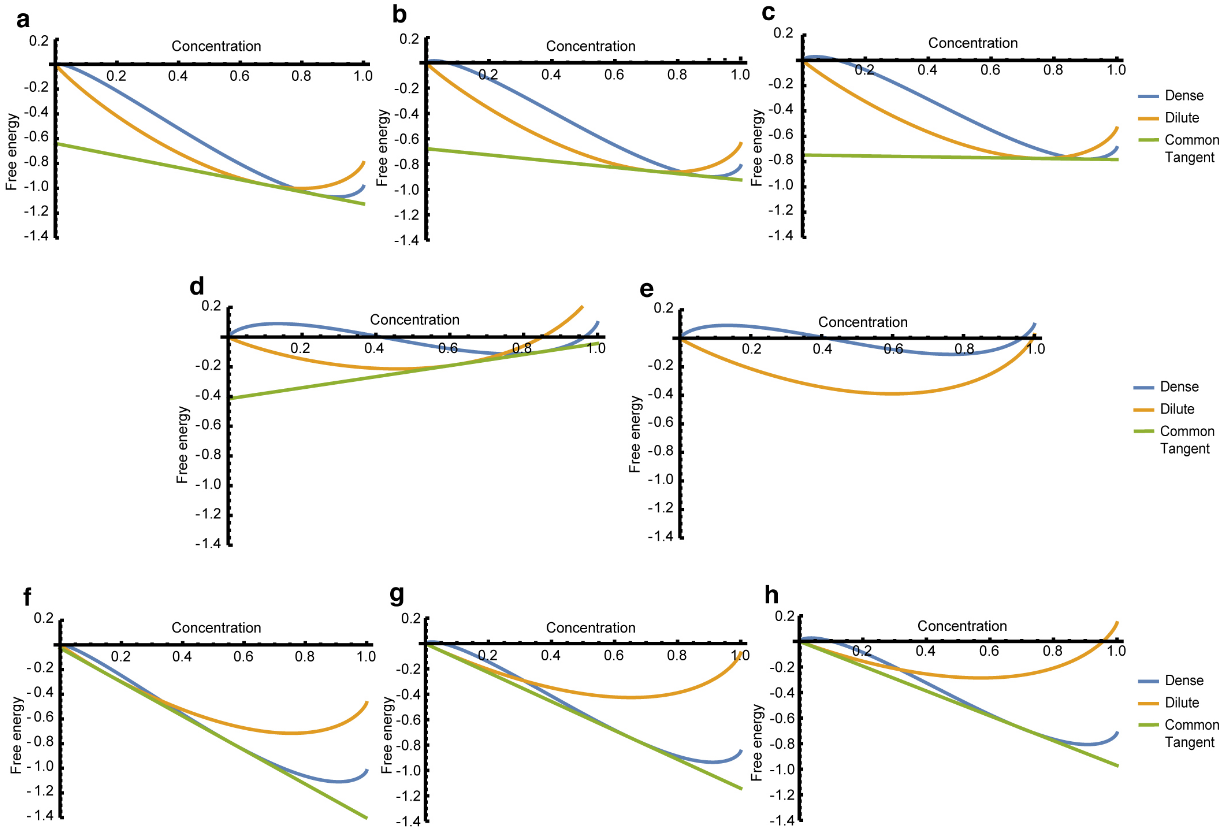

Supplementary Figure 8. Free energies of the analytical models in the dilute (orange) and dense (blue) limits of **a** A_4:B_4, **b** A_6:B_6, **c** A_8:B_8, **d** A_8:B_{8U}, **e** A_8:B_{8R}, **f** A_3:B_4, **g** A_5:B_6, and **h** A_7:B_8 systems.

Supplementary References

[1] P. Li, S. Banjade, H.-C. Cheng, S. Kim, B. Chen, L. Guo, M. Llaguno, J. V. Hollingsworth, D. S. King, S. F. Banani, P. S. Russo, Q.-X. Jiang, B. T. Nixon, and M. K. Rosen, Nature **483**, 336 (2012).

[2] Y. Lin, D. S. W. Protter, M. K. Rosen, and R. Parker, Mol. Cell **60**, 208 (2015).

[3] S. F. Banani, A. M. Rice, W. B. Peeples, Y. Lin, S. Jain, R. Parker, and M. K. Rosen, Cell **166**, 651 (2016).

[4] L. C. M. Mackinder, M. T. Meyer, T. Mettler-Altmann, V. K. Chen, M. C. Mitchell, O. Caspari, E. S. Freeman Rosenzweig, L. Pallesen, G. Reeves, A. Itakura, R. Roth, F. Sommer, S. Geimer, T. Muhlhaus, M. Schroda, U. Goodenough, M. Stitt, H. Griffiths, and M. C. Jonikas, Proc. Natl Acad. Sci. USA **113**, 5958 (2016).

[5] A. N. Semenov and M. Rubinstein, Macromolecules **31**, 1373 (1998).

[6] T. S. Harmon, A. S. Holehouse, M. K. Rosen, and R. V. Pappu, eLife **6**, e30294 (2017).

[7] P. J. Flory, *Principles of Polymer Chemistry* (Cornell University Press, 1953).
